# Scrophulariae Radix-Atractylodes sinensis pair and metformin inhibit inflammation by modulating gut microbiota of high-fat diet/streptozotocin-induced diabetes in rats

**DOI:** 10.3389/fmicb.2022.900021

**Published:** 2022-11-30

**Authors:** Xiaoxia Guo, Chong Wang, Ranran Zhang, Xuliang Hao, Lei Lv, Yan Ni, Xiaohong Fan, Weiliang Zhang, Yunhong Jiao, Wei Song, Qi Dong, Yuqi Qi, Meiqing Song, Xuemei Qin

**Affiliations:** ^1^Department of Metabolism, Shanxi Institute of Traditional Chinese Medicine, Taiyuan, Shanxi, China; ^2^Traditional Chinese Medicine Preparation Center, Affiliated Hospital of Shanxi University of Chinese Medicine, Taiyuan, Shanxi, China; ^3^Clinical Pharmacological Research Laboratory, Shanxi Institute of Traditional Chinese Medicine, Taiyuan, Shanxi, China; ^4^Modern Research Center for Traditional Chinese Medicine, Shanxi University, Taiyuan, Shanxi, China

**Keywords:** type 2 diabetes mellitus, Scrophulariae Radix, Atractylodes sinensis pair, metformin, gut microbiota

## Abstract

**Introduction:**

Type 2 mellitus (T2DM), a chronic metabolic disorder, causes severe impairment of patients’ quality of life and has attracted global attention. Many studies have suggested the importance of the gut microbiota in the occurrence of T2DM. The Scrophulariae Radix and Atractylodes sinensis (XC) pair, recommended in traditional Chinese medicine (TCM), have been used for treating diabetes for many years. However, research on the role of the XC pair in modulating gut microbial communities is lacking, but it is important to elucidate the underlying mechanism.

**Methods:**

In this study, we detected bacterial communities by high-throughput 16S rRNA gene sequencing.

**Results:**

The results showed that XC + MET reduced postprandial hyperglycemia and inflammatory response in diabetic rats more effectively than metformin (MET) alone. The XC + MET treatment reshaped the intestinal microbial composition of diabetic rats. XC can help MET regulate carbohydrate, amino acid, and lipid metabolism, particularly the insulin signaling pathway.

**Discussion:**

This research would help elucidate potential mechanisms and the treatment methods.

## Graphical Abstract

The graphic abstract of the study. The figure was created with BioRender.com. 
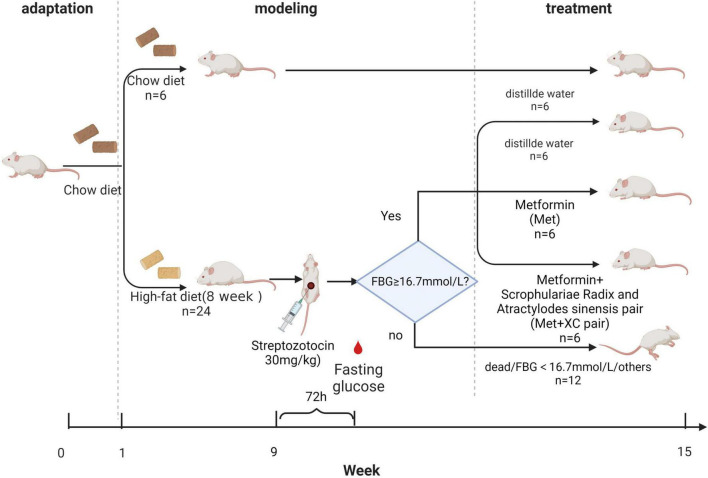

## Introduction

Type 2 diabetes mellitus (T2DM) is a chronic degenerative metabolic disorder characterized by long-term hyperglycemia and the pathophysiological feature of insulin resistance (IR) (Nathan, 2015). The prevalence of glucose tolerance impairment and diabetes has recently increased among adults worldwide, with 90% of these people having T2DM (Guariguata et al., 2014; Tong et al., 2018; Magliano et al., 2019). The majority of diabetes cases are reported in China (Li et al., 2020). Diabetes is a major global health concern due to its high prevalence, as well as the associated mortality and disability (Baena-Díez et al., 2016; Saeedi et al., 2020). Thus, diabetes prevention and treatment have been extensively researched over the last few decades, and diabetes is a global concern. T2DM is primarily caused by obesity, which is associated with IR and mild inflammation (Navab et al., 2008; Bakkar et al., 2020). According to earlier research, the gut microbiota can cause these diseases and has a significant impact on the pathophysiology of diabetes (Van Olden et al., 2015; Leustean et al., 2018; Xiao et al., 2021).

Metformin (MET) is the oral medicine recommended for the initial treatment of T2DM. It exhibits anti-diabetic effects by changing gut microbial communities (Cherney and Lam, 2018; Guo and Xie, 2018; Sun et al., 2018; Vallianou et al., 2019). MET has multiple activities and excellent therapeutic efficacy in different pathological environments (Das et al., 2021; Drzewoski and Hanefeld, 2021; Rajgopal and Kochhar, 2021). However, most patients under long-term MET treatment (>10 years) have poor blood glucose control (Cherney and Lam, 2018). Since no drug has a better overall glycemic response, a majority of patients eventually need additional oral drugs and insulin to maintain glucose homeostasis while reducing diabetes-related complications (Strain et al., 2020).

Traditional Chinese medicine (TCM) is an important source for developing anti-diabetic agents, which are generally used as an effective complementary and alternative medicine to ameliorate diabetes along with conventional anti-diabetic drugs (Shin et al., 2017; Tong et al., 2018; Wei et al., 2018). The medical application of the Scrophulariae Radix and Atractylodes sinensis (XC) pair was introduced by Shi Jinmo, the sage of TCM, which has been administered in the clinic combined with anti-diabetic drugs for over 30 years and has shown satisfactory therapeutic effects. Scrophulariae Radix is a herbal medicine containing harpagide, which possesses several beneficial pharmacological effects, like suppressing inflammation, enhancing immunity, and inhibiting apoptosis (Sheu et al., 2015; Lee et al., 2021). Atractylodes sinensis, containing atractylodin and quinic acid, may ameliorate hyperglycemia, hyperlipidemia, and IR, downregulate inflammatory cytokines and improve gut microbial imbalance (Arya et al., 2014; Pang et al., 2021). In the previous experiment, we studied the hypoglycemic effect of XC on high-fat diet (HFD)/streptozotocin induced diabetic rats. However, XC has not been found to have a significant hypoglycemic effect. Therefore, we speculated that XC may be used as an auxiliary drug to enhance the hypoglycemic effect of MET. So far, the therapeutic efficacy of XC combined with MET and their possible beneficial synergy in the gut microbiota for diabetes has not been investigated. Therefore, the aim of this study was to examine whether XC + MET would improve glucolipid metabolism and modulate gut microbiota in diabetic rat.

## Materials and methods

### Preparing the Scrophulariae Radix and Atractylodes sinensis herbal formula

The XC pair contained Scrophulariae Radix and Atractylodes sinensis. All herbs were obtained from (Jiangyin Tianjiang Pharmaceutical Co., Ltd., Jiangsu, China) and their quality was controlled. The herbal medicines used for making the boil-free granules were from the same place and the same batch, and they were taken twice daily after mixing. The production of the boil-free granules for each herb involved four steps. (1) Each herb was boiled with water twice to extract the major components. (2) The decoction of each herb was combined and filtered with 200-mesh. (3) The filtrate of the different herbs was concentrated in pasty extracts with different relative densities at 50°C. (4) The extracts were converted to granules using spray drying and were packaged. Supplementary Table 1 shows the quantity of the granular extract for every herb in the daily effective amount of XC used clinically by diabetic patients. The samples were stored at 4°C.

The major chemical components of XC were detected and identified by the Institute of Chinese Materia Medica from the Shanxi Provincial Institute of Traditional Chinese Medicine. All the herbs met the Chinese Pharmacopeia standard (Zhang et al., 2015) following the determination method in the “Chinese Pharmacopeia (2015 edition).” We conducted high-performance liquid chromatography (HPLC) to evaluate the stability and quality of the three batches of herbal formula granules. We also used a reverse-phase C18 column (100 mm × 2.1 mm, 1.8 μm) to separate chromatographic fractions, and its mobile phase contained acetonitrile (A) −0.1% formic acid aqueous solution and (B) at a column temperature of 30°C and flow rate of 0.4 mL/min. Fingerprints of Scrophulariae Radix, Atractylodes sinensis, and their constituent herbs were separately obtained using an optimized method. Three major components in the formula of the XC pair were identified at the detection wavelength of 284 nm.

### Constructing the diabetic rat model

We obtained 36 healthy male SD rats weighing 200 ± 20 g from Huafukang Biotechnology Co., Ltd. (Beijing, China; License, No. SCXK (Beijing) 2019-0,008). All animals were raised under specific pathogen free (SPF) conditions. The rats were acclimated for 1 week at 25°C and 50–60% humidity following a 12 h light/dark cycle. They could eat and drink freely. Our study protocols were approved by the Research Ethics Committee of the Shanxi Provincial Institute of Traditional Chinese Medicine (Taiyuan, China). All experiments were performed following the Guide for the Care and Use of Laboratory Animals.

After 1 week of adaptive feeding in an SPF environment, 30 rats were fed a HFD (60% fat + 20% carbohydrate + 20% protein) sterilized under ultraviolet radiation (Furman, 2021). After eight consecutive weeks of this regimen, 24 rats (six were adopted for supplementing rats failing in model construction) were intraperitoneally injected with 30 mg/kg Streptozotocin (STZ) (dissolved in 0.1 M sterile citric acid buffer, pH = 4.3; Sigma-Aldrich Ltd., Shanghai, China). Three days after injection, fasting blood glucose (FBG) levels were tested, and rats with FBG levels above 16.7 mmol/L were considered diabetic rats (Wu et al., 2019). Two rats died of hyperglycemia. Six rats were excluded because their FBG did not reach the threshold. We randomly selected 18 rats from 22 rats with FBG greater than 16.7 mmol/L for treatment. The 18 diabetic rats were divided into three groups by the random number table method. One group was treated with MET (*n* = 6, MET group), the second group was treated with XC + MET (*n* = 6, XC + MET group), and the other group was treated with water (*n* = 6, model group). In addition, we also measured the FBG of the three groups to ensure that there was no significant difference in the initial FBG between the three groups. In the normal control group, six rats were fed a normal pellet diet and injected with the same volume of sterile citric acid buffer.

### Experimental design

In this study, three treatment groups of rats were constructed. The rats in the MET group were administered 200 mg/kg/day MET (Cherney and Lam, 2018), those in the XC + MET group were administered 810 mg/kg/day XC and 200 mg/kg/day MET, and those in the diabetes group were administered water treatment. Additionally, the rats in the normal group were also administered water treatment. We determined these dosages by equivalently converting the measurement of the body surface area (BSA) in animals to that of humans. All treatments were administered orally, with water used as a vehicle. The rats were treated for 6 weeks, followed by overnight (12 h) fasting. Then, blood was taken from the orbit. After blood collection, the rats were given 50% glucose solution by gavage with 2 ml/kg an oral glucose tolerance test (OGTT). Two hours later, all rats were injected with chloral hydrate for anesthesia, and blood was collected through an abdominal aortic puncture. The blood was centrifuged at 4,000 rpm and 4°C for 10 min to separate the serum and then stored at −80°C for analysis. We also collected fresh stool samples and preserved them at −80°C.

### Biochemical analysis

We determined the weight of the rats, drinking water, and food intake of rats every week and collected blood from the tail tip weekly for measuring FBG using the glucose meter (ONETOUCH, Ultra, LifeScan, Shanghai, China). Before blood collection, all rats were fasted for 6–8 h (from 7:00 a.m. to 1:00–3:00 p.m.). The levels of low-density lipoprotein cholesterol (LDL-C), total cholesterol (TC), blood urea nitrogen (BUN), alanine aspartate aminotransferase (AST), aminotransferase (ALT), 2-h postprandial blood glucose (PBG), and fasting plasma glucose (FPG) were determined using an automated biochemical analyzer (Au640, Olympus, Japan). The fasting serum insulin (FINS) and glycosylated serum protein (GSP) levels were also measured using kits (Nanjing Jiancheng Bioengineering Institute, China). The NLRP3 level was measured using rat ELISA kits (Enzyme-linked Biotechnology Co., Ltd., Shanghai, China). Additionally, the formula HOMA-IR = FINS × FBG/22.5 was used to determine the Homeostasis Model Assessment-IR (HOMA-IR) index.

### Stool sampling and DNA isolation

Stool samples were collected on examination and stored immediately at −80°C. Then, the Fast DNA SPIN extraction kit (MP Biomedicals, Santa Ana, CA, United States) was used for extracting and purifying DNA following specific protocols. Then, the purified DNA samples were stored at −20°C for further analysis. The quality and content of the isolated DNA were determined by agarose gel electrophoresis (AGE) and using the NanoDrop ND-1000 spectrophotometer (ThermoFisher Scientific, Waltham, MA, United States), respectively.

### PCR amplification and sequence analysis

Using the primers 338F (5′-ACTCC TACGGGAGGCAGCA-3′, Forward) and 806R (5′-GGACTACHVGGGTWTCTAAT-3′, Reverse), the V3–V4 region of bacterial 16S rRNA genes was amplified through PCR. Briefly, the PCR was performed following a previously reported method (Li et al., 2014). Following amplification, Agencourt AMPure beads (Beckman Coulter, Indianapolis, IN, United States) were used for purifying PCR amplicons, and the PicoGreen dsDNA Assay Kit (Invitrogen, Carlsbad, CA, United States) was used for purification. Amplicons of equivalent volumes were combined after quantification. Then, the MiSeq Reagent Kit v3 in the Illumina MiSeq platform was used for pair-end 2 bp × 300 bp sequencing by Shanghai Personal Biotechnology Co., Ltd. (Shanghai, China).

As previously described, the sequencing data were processed using the Quantitative Insights into Microbial Ecology (QIIME, v1.8.0) pipeline (Caporaso et al., 2010). Following chimera discovery, we used the UCLUST algorithm to cluster all high-quality sequences into operational taxonomic units (OTUs) based on 97% sequence identity (Edgar, 2010). Following that, one typical sequence with default parameters was chosen for each OTU. The BLAST algorithm was then used to search typical sequence sets in the Greengenes database (DeSantis et al., 2006) for OTU taxonomy classification using the best hit method (Altschul et al., 1997). We created an OTU table to keep track of the OTU levels in each sample and taxonomic classification. OTUs with <0.001% of the total sequences in the samples were removed. We created the averaged, rounded, rarefied OTU table by taking the average of 100 uniformly resampled OTU subsets at 90% of minimal sequencing depth for minimizing different sequencing depths across samples.

MiSeq raw sequences from 24 rat fecal samples have been submitted to the National Center for Biotechnology Information (NCBI) Project under accession number PRJNA818640.

### Estimation of gut microbial metagenomic functional levels

The eligible 16S rRNA sequences were aligned and annotated using the Greengenes database’s pre-set 97%-level OTU (DeSantis et al., 2006). To reduce the impact of sequencing errors, non-singleton sequences were first aligned against Greengenes, and then reference sequences that matched one or more times were obtained for forming the novel database. The closed-reference OTUs were chosen from Greengenes using the global alignment algorithm USEARCH (Edgar, 2010). The OTU table was normalized using the sequencing depth, and the related functional genes were predicted using the PICRUSt software package (Morgan et al., 2013). Finally, we searched the Kyoto Encyclopedia of Genes and Genomes (KEGG) database for predicted genes (Kanehisa et al., 2008). We used a web-based program^1^ to conduct LEfSe (linear discriminant analysis effect size) analyses to identify taxa with different relative abundances among diverse groups. (Segata et al., 2011) under the following conditions: logarithmic linear discriminant analysis (LDA) score of differential characteristic selection threshold >2.0, and factorial Kruskal–Wallis test across diverse classes with α < 0.05.

### Statistical analysis

The R statistical program (version 3.1.0) was used for statistical analysis. Following the normal distribution and variance homogeneity testing, one-way Analysis of Variance (ANOVA) was performed for multiple groups, followed by Tukey correction (GraphPad 9.0, La Jolla, CA, United States). To make a statistical difference in the abundance of the genera that failed the normality test, the Kruskal–Wallis *H* test was used. The data were expressed as the interquartile range’s median. The two-tailed Pearson’s correlation analysis was performed using the OmicStudio tools at https://www.omicstudio.cn/tool. A *P*-value of less than 0.05 was used as the significance threshold.

## Results

### The chemical composition in Scrophulariae Radix and Atractylodes sinensis pair

The results of the HPLC analysis showed that the XC pair contained three compounds, including harpagide, harpagoside, and quinic acid. As expected, atractylodin was not detected because it was a fat soluble component. The harpagide content of Scrophulariae Radix was 2.378 μg/mg, Harpagoside content was 1.009 μg/mg, and the quinic acid content of Atractylodes sinensis was 0.323 μg/mg. The total ion chromatogram and the results of XC samples are shown in Supplementary Figure 1 and Supplementary Tables 2, 3.

### The effect of XC + MET on the body weight and metabolic factors of diabetic rats

**FIGURE 1:**
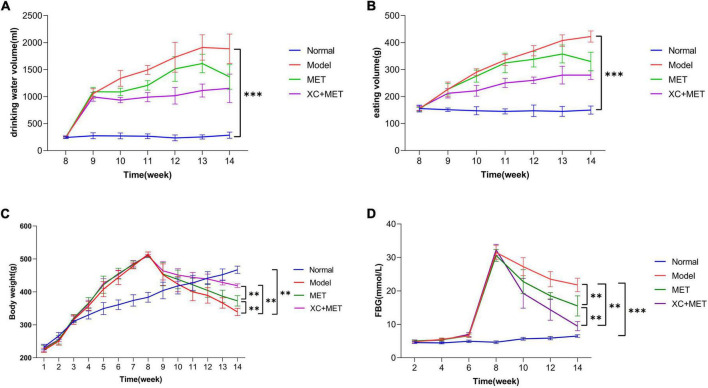
The effect of EMT + XC on the drinking water, eating volume, BW, and fasting blood glucose (FBG) of diabetic rats. **(A)** Drinking water volume, **(B)** eating volume, **(C)** body weight (BW), and **(D)** FBG. Blue, red, green, and purple lines indicate normal, model, metformin (MET)-treated, and XC + MET-treated diabetic rats, respectively. Data are presented as the mean ± SD, *n* = 6; ^**^P < 0.01 and ^***^P < 0.001.

The results suggested that the hair of the rats in the normal group became smooth and bright with age, with favorable mental state and development, fast reflexes, stable water intake and urine output during experimental period, and their FBG was between 3.0 and 5.0 mmol/L. During the experiment, the rats in the diabetic group get fat gradually, became thinner after injection of STZ, and developed hair disordered, and were inactive and irritable, with considerably higher water and food consumption, as well as urine volume (Figures 1A,B). The diabetic rats exhibited the representative diabetic symptoms, including “more feeding, drinking, urine passage, and less weight.” Figure 1D shows the FBG levels of the different rat groups. The diabetic rats had significantly higher FBG (>16.7 mmol/L) than the rats in the normal control group, which indicated that the diabetic rat was successfully constructed after STZ induction. The XC + MET-treated rats had considerably lower FBG and significantly lower body weight (BW) than the MET-treated rats (Figures 1C,D).

Moreover, we also evaluated the metabolic parameters in the different treatment groups. The expression levels of TC, LDL, FPG, PBG, HOMA-IR, GSP, liver weight/BW, and NLRP3 in the diabetes group were significantly higher after the modeling was completed (*P* < 0.05; Supplementary Figure 2). However, after treatment for 6 weeks, the rats in the MET and XC + MET groups had substantially lower serum LDL, FPG, PBG, GSP, and HOMA-IR concentrations (*P* < 0.05) relative to those in the diabetes group. Serum FINS (*P* = 0.33), TC (*P* = 0.57), ALT (*P* = 0.48), BUN (*P* = 0.18), and liver weight/BW (*P* = 0.94) were similar in the MET and XC + MET groups. NLRP3 expression was substantially lower in the XC + MET-treated rats but not in the MET-treated rats when compare with untreated diabetic rats. The results indicated that XC or MET could synergistically influence dyslipidemia, hyperglycemia, and IR. Moreover, XC could enhance the anti-inflammatory effect of MET. The values of ALT and BUN also showed that XC could alleviate liver and kidney toxicity caused by diabetes, although the effect was not significant (*P* > 0.05).

### Alteration in the gut microbiota by metformin and Scrophulariae Radix and Atractylodes sinensis

**FIGURE 2:**
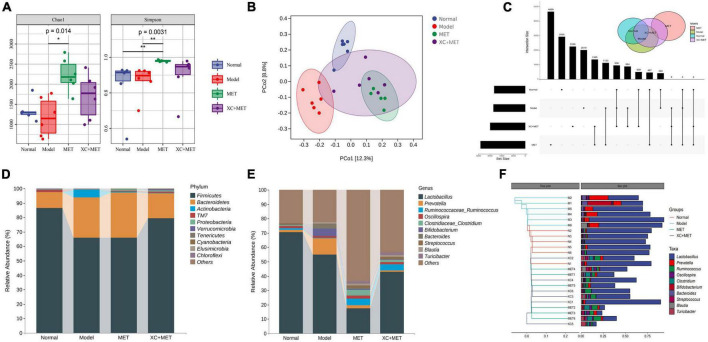
XC + MET regulated gut microbial composition and structure in the model rats. The gut microbial communities of the normal, model, metformin (MET), and XC + MET groups. **(A)** Box plots indicate the different microbial diversities based on the operational taxonomic unit (OTU) quantity, the Chao1 index, and the Simpson index, respectively. Significant differences were confirmed by performing Dunnett’s test and the Kruskal–Wallis rank-sum test; *P < 0.05 and **P < 0.01. **(B)** The principal coordinates analysis (PCoA) is based on the unweighted UniFrac distance, with an ellipse confidence of 0.95. **(C)** The amplicon sequence variants (ASV) Venn diagram. **(D,E)** The gut microbial compositions according to genera and phyla. **(F)** Hierarchical clustering analysis of gut microbial communities based on genera. The hierarchical clustering tree is presented on the left, while the stacked column chart of genera is displayed on the right.

To determine the relationship between the effect of XC + MET and the alterations of gut microbial communities, which critically influence the occurrence of diabetes, we collected the fecal samples of the rats and pair-end sequenced gut microbial 16S-V3V4 regions using the Illumina high-throughput sequencing platform. We obtained 1,681,358 sequences after denoising, and 1,058,585 high-quality sequences were obtained from 24 samples, i.e., 44,107 ± 5,241 reads were obtained for every sample. Individual rarefaction curves showed that each sample attained great sampling coverage (Supplementary Figure 3). Significant differences were detected across the four groups based on alpha-diversity indices. Overall, the model group had significantly lower diversity than the normal group. Both Chao1 and Simpson indices in MET or XC + MET rats were significantly higher than those of the model rats (Figure 2A). We also conducted principal coordinates analysis (PCoA) on the unweighted UniFrac distances to compare microbial structures under three conditions. The results indicated that the gut microbial structures differed among the four groups. The three principal components (PCs) occupied 33.08, 19.04, and 15.55% of the overall variation (Figure 2B). Thus, the shared amplicon sequence variants (ASV) between the model and normal groups was 178, and that between the model and MET groups and the model and XC + MET-treated groups increased to 196 and 187, respectively (Figure 2C), indicating that both the intervention of MET and XC + MET probably altered the gut microbial composition in rats, which is consistent with the previous results of microbial diversity.

The alterations in the gut microbial composition were also examined. The 10 most abundant phyla and genera within the fecal microbial communities in the different groups are shown in Figures 2D,E. From the tree, the gut microbial communities were found to include sequences of six bacterial phyla/divisions. Many sequences were associated with Bacteroidetes and Firmicutes, while the remaining sequences were of bacteria belonging to TM7, Verrucomicrobia, Proteobacteria, and Actinobacteria. Among all the phyla recorded, Firmicutes had the highest abundance in all the samples. The model rats had lower Firmicutes abundance but higher Bacteroidetes abundance than normal rats (Figure 2D). Compared with model rats, only XC + MET seemed to increase Firmicutes and reduce Actinobacteria and Bacteroidetes in Supplementary Figure 4 (*P* < 0.05). MET alone does not appear to have this effect. The model rats had a lower Firmicutes-to-Bacteroidetes (F/B) ratio than the rats in the normal group. Comparion to the normal group, the F/B ratio seems to remain low after MET treatment. While a higher ratio was observed in the XC + MET-treated rats than MET alone (Supplementary Figure 5). Which indicates XC could rectify the decrease of F/B ratio caused by MET. According to taxon-based analyses, XC could assist MET-altered the microbial composition relative to that in the diabetic group.

The six most significantly abundant phyla along with the 10 most significantly abundant genera within the fecal microbial communities in the different treatment groups are presented in Figures 2D,E. The 10 most abundant gut microbial genera were analyzed by hierarchical clustering, which showed that the abundance of the other genera in the MET and XC + MET-treated groups were similar to those of the normal group except *Lactobacillus* (Figure 2F). The abundance of *Lactobacillus* in the model group was significantly lower than that in the normal group. Interestingly, the lower abundance of *Lactobacillus* after MET treatment increased significantly in the XC + MET group. The results showed that XC could antagonize the inhibitory effect of MET on *Lactobacillus*.

### Scrophulariae Radix and Atractylodes sinensis modulated the biomarker levels of the gut microbes in the diabetic rats

**FIGURE 3:**
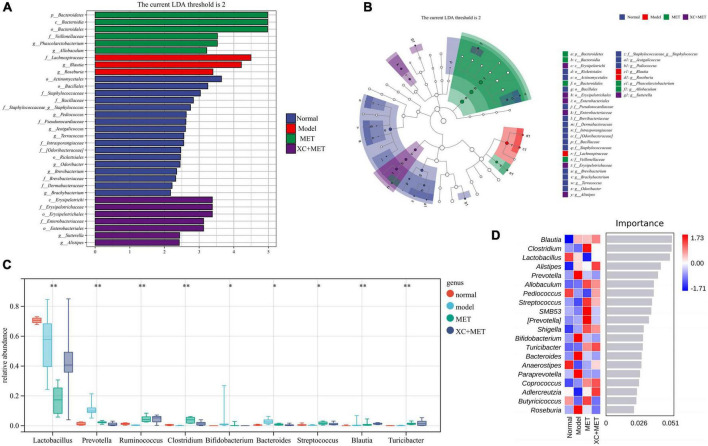
The linear discriminant analysis (LDA) and LEfSe (linear discriminant analysis effect size) were conducted based on the different operational taxonomic units (OTUs) of the gut microbial communities in the normal, model, metformin (MET), and XC + MET groups. The taxa with the highest differential abundances among the four groups were identified. **(A)** The LDA scores of the microbial communities were significantly different across the four groups. **(B)** The taxonomic cladogram was constructed by performing the LEfSe analysis. Blue, red, green, and purple indicate taxa enriched in the normal, model, MET, and XC + MET groups, respectively. LDA, linear discriminant analysis; LEfSe, linear discriminant analysis effect size. **(C)** The abundances of different microbial genera across the four groups. The level of significance was determined by performing Wilcoxon’s signed-rank test; *P < 0.05, ^**^P < 0.01. **(D)** The random forest analysis for differentially abundant gut microbial communities. The color intensity indicates the gut microbial distribution of every sample (red and blue indicate high and low abundances, respectively).

To detect the genus-level biomarkers, we conducted comparisons across four groups of the gut microbial communities. The abundances of five genera were higher, and six genera were lower in diabetic rats relative to their abundances in the rats of the normal group (Supplementary Table 4). Compared with diabetes rats, the abundance of 11 genera was higher and 4 genera were lower in MET-treated rats (Supplementary Table 5). Interestingly, seven genera were higher and seven genera were lower in XC + MET-treated rats. Among them, the abundance of *Bifidobacterium*, *Paraprevotella*, *Bacteroides*, *Prevotella*, and *Phascolarctobacterium* was lower, while *Clostridium*, *Dehalobacterium*, and *Ruminococcus* higher in the XC + MET-treated rats (Supplementary Table 6). Compared with MET-treated rats, the abundance of *Pediococcus* was higher, while the abundance of *Butyricicoccus* and SMB531 was lower in the XC + MET group than that in the MET group (Supplementary Table 7). These results suggested that gut microbiota composition was differentially modulated in the diabetic rats that were administered XC + MET treatment. The significantly different microbial communities, together with corresponding taxonomic hierarchies, are shown as LDA distribution histograms and a related cladogram of the rats in the four groups (Figures 3A,B). The abundances of the MET and/or XC interventional biomarkers are presented in Figure 3C. The cladogram based on the LEfSe results showed that the abundance of nine gut microbial communities differed significantly at the genus level across the four groups. A random forest analysis was performed to determine their order of importance. *Clostridium*, *Lactobacillus*, *Alistipes*, and *Prevotella* played dominant roles in determining the differences across the various treatment groups (Figure 3D).

### Correlation of microbial regulation with biochemical parameters

We performed a correlation heat map analysis to determine the correlation between the intestinal microbiota and diabetes related glucose-metabolism parameters (BW, Liver/BW, FINS, FPG, GSP, HOMA-IR, PBG, and NLRP3).

The Liver/BW was positively correlated with *Blautia* and *Bacteroides* but negatively correlated with the F/B ratio. The FINS levels were positively correlated with *Lactobacillus* and negatively correlated with *Blautia*, suggesting that *Blautia*, *Bacteroides*, *Lactobacillus*, and F/B ratio play an important role in the pathogenesis of diabetes (Supplementary Figure 6).

The PBG level was positively correlated with *Blautia* and significantly negatively correlated with *Dehalobacterium*. NLRP3 was positively correlated with *Blautia*. The BW was positively correlated with the F/B ratio and negatively correlated with *Bacteroides*. FPG was positively correlated with *SMB53* and negatively correlated with *Pseudomonas*, and HOMA-IR was positively correlated with *SMB53*. There was no significant correlation between GSP and critical intestinal microbiota (Supplementary Figure 6). It is suggested that *Blautia*, *Dehalobacterium*, *Pseudomonas*, *SMB53*, F/B ratio, and *Bacteroides* play a crucial role in the treatment of XC and/or MET (Supplementary Figure 6).

### Functional prediction of gut microbial communities of the four groups

**FIGURE 4:**
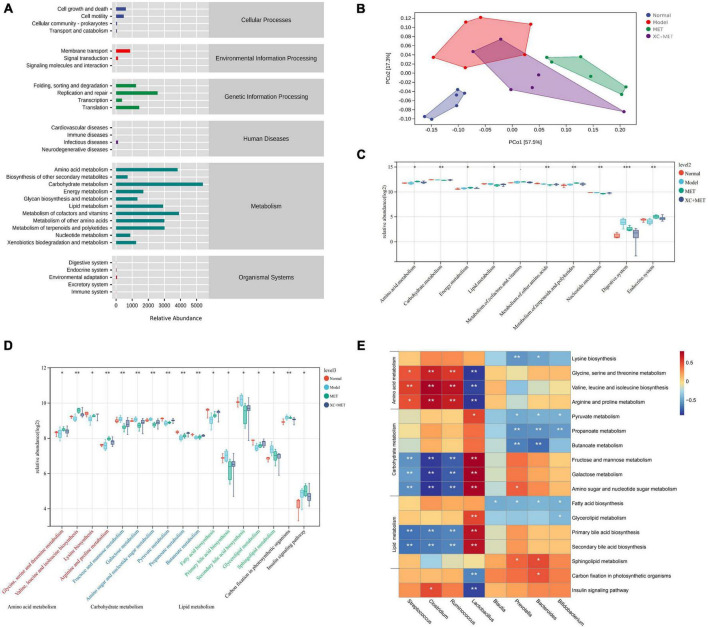
The changes in the diabetic rats treated with metformin (MET) and Scrophulariae Radix and Atractylodes sinensis (XC). **(A)** Enrichment of the Kyoto Encyclopedia of Genes and Genomes (KEGG) pathways for the gut microbial communities of the rats at the secondary classification level. **(B)** Principal coordinates analysis (PCoA) of the functional units of gut microbial communities according to Bray–Curtis similarity, with an ellipse confidence of 0.95. **(C)** The altered KEGG functional pathway abundances after XC and/or MET treatment of level 2. **(D)** The altered KEGG functional pathway abundances after XC and/or MET treatment of level 3. The KEGG pathway enrichment is displayed as the mean ± SD; *P < 0.05, **P < 0.01, and ***P < 0.001 based on Wilcoxon’s signed-rank test. **(E)** The correlation between the microbial communities and the KEGG functional pathways. The color intensity indicates correlation strength (red and blue indicate positive and negative correlation, respectively); *P < 0.05, **P < 0.01, and ***P < 0.001.

To further elucidate the association of gut microbial communities with XC + MET treatment, we examined the microbial metabolic activities through metagenomic analysis. By using the PICRUSt software package, metagenome functions were predicted by detecting the 16S rRNA genes (Morgan et al., 2013). Then, we collapsed the metagenomes against the KEGG database of level 2. The gut microbial functions of all rats were mostly related to metabolism, especially for the metabolism of carbohydrates, lipids, and amino acids (Figure 4A). As suggested by the PCoA, microbial functions were slightly separated across the four groups. In the PC1 dimension, the MET and XC + MET groups had close functional composition compared to that in the normal group, and the contribution rate was 57.5% (Figure 4B). XC and MET elevated the levels of three metabolic processes relative to their levels in the model group; XC + MET could significantly improve the levels of the first two metabolic processes. We then collapsed the metagenomes to the KEGG database of level 3. The results showed that 17 pathways were significantly different among the four groups, including the insulin signaling pathway and the metabolism of the three aforementioned substances (Figure 4C).

To determine the association of gut microbial communities with metabolites, we performed a clustering correlation analysis of eight genera in the gut microbiota and 17 markedly altered metabolites in feces. A heat map was constructed to display gut microbial communities’ negative and positive correlations with identified metabolites (Figure 4D). The abundances of *Streptococcus*, *Clostridium*, and *Ruminococcus* were positively related to amino acid metabolism but significantly negatively related to carbohydrate and lipid metabolism. *Prevotella*, *Bacteroides*, and *Bifidobacterium* showed a significantly negative correlation with amino acid, carbohydrate, and lipid metabolism. Additionally, *Lactobacillus* showed a significantly positive relationship with carbohydrate and lipid metabolism and a significantly negative relationship with amino acid metabolism and the insulin signaling pathway (Figure 4E).

## Discussion

Gut microecology keeps an individual healthy and prevents disorders (O’Keefe, 2008). Altering the gut microbial composition and function can increase the level of inflammatory reactions, reduce insulin sensitivity, and increase diabetes susceptibility (Cani et al., 2008; Boulangé et al., 2016). The cross-effect of gut microbial communities, systemic inflammation, and IR is important to elucidate the specific mechanisms related to diabetes-associated disorders. Therefore, regulating the intestinal flora and improving the inflammatory response are important for the prevention and treatment of diabetes.

In this study, we investigated whether the gut microbial community can serve as the pharmacological target for reducing blood glucose levels when XC is administered orally to diabetic rats with MET. STZ was first used in animal models of type 1 and type 2 diabetes in the mid-1960s (Deeds et al., 2011; Eleazu et al., 2013). Because STZ alone cannot effectively mimic diabetes, researchers are now using an HFD regimen to induce IR and hyperinsulinemia in animals before injecting them with STZ (Furman, 2021). This leads to the classic diabetes symptoms of overeating, polyuria, polydipsia, and insulin deficiency. The technique assisted us in determining the relationship between diabetes and XC treatment and gut microbial communities. After 14 weeks, we discovered that XC + MET had the same effect on diabetic rats’ blood FPG, GSP, and LDL levels as MET (Nasri and Rafieian-Kopaei, 2014). Furthermore, the XC + MET combination treatment improved weight loss, hyperglycemia, lipid metabolism disorder, IR, and diabetes-related inflammasome. Interestingly, a significant reduction in PBG was observed in the XC + MET-treatment group, indicating that XC was important in assisting MET in regulating PBG levels in diabetic rats. Hyperglycemia after meals is the therapeutic target for diabetes patients, and numerous guidelines on post-prandial glycaemia (PPG) control have been issued (American Diabetes Association, 2011). PPG, in particular, can predict the risk of diabetes-related complications independently (Mannucci et al., 2012). We found no statistical differences in FINS and TC levels after MET or XC + MET therapy, which was likely due to the shorter course of treatment in this study compared to other studies. Therefore, studies with a longer course of treatment are required.

Diabetes and IR are influenced by NLRP3 levels, which could be used as anti-diabetes treatment targets (Stienstra et al., 2011). High glucose levels can activate the NLRP3 inflammatory body, which is linked to metabolic diseases like diabetes and obesity (Dixit, 2013; Wan et al., 2019; Rai et al., 2020). More importantly, we discovered that NLRP3 expression was significantly increased in diabetic rats but significantly decreased after XC + MET exposure, implying that XC + MET can downregulate the activation of NLRP3 inflammatory corpuscles in diabetic rats. We also looked at how NLRP3 expression correlated with ALT, BUN, FBG, PBG, liver weight/BW, GSP, and FINS levels. The level of NLRP3 was found to be negatively correlated with FINS but significantly positively correlated with other factors. Therefore, more research is needed to determine the role of XC in NLRP3-related signaling pathways.

The intestinal microbial diversity of HFD/STZ-induced diabetic rats decreased as inflammation, insulin levels dropped, and IR emerged, which is consistent with previous research (Ling et al., 2014). After XC + MET treatment, the gut microbial diversity improved. It is worth mentioning that both MET and XC + MET appear to increase gut microbial diversity to levels exceeding that of control rats. This would imply that species that are usually not very prominent become more prominent after MET and XC + MET treatment. Some studies have shown that MET has additional health benefits for T2DM patients/rats, which is related to gut microbial changes (Cabreiro et al., 2013; Pryor et al., 2019; Mueller et al., 2021). We speculate that the increase of gut microbial diversity in MET-treatment in this study may be related to the gastrointestinal reaction induced by MET. However, the intervention of XC reduces the gastrointestinal reaction and leads to the decrease of gut microbial diversity. Of course, we need further research to clarify the mechanism of this change. Based on the findings, we found that there were some clinically significant phylum and genus-level differences after XC + MET treatment. We hypothesized that the gut microbiota was the pharmacological target for the hypoglycemic effect of XC + MET after oral administration to diabetic rats. Bacteroidetes and Firmicutes were the phyla with the highest abundance in all samples. Firmicutes are gram-negative bacteria that dominate the human intestinal flora. According to the data, the relative abundance of Firmicutes decreased significantly while that of Bacteroidetes increased slightly in diabetic rats compared to non-diabetic rats (Larsen et al., 2010). *Bacteroides* abundance was significantly higher in the MET group than in the diabetes group but lower in the XC + MET group, indicating that XC can assist MET in inhibiting the increase of *Bacteroides* caused by diabetes. MET causes weight loss in overweight and obese patients (Seifarth et al., 2013), which is most likely due to the abundance of *Bacteroides* (Ley et al., 2006). The F/B ratio can be used as a gut microbial health indicator (Tseng and Wu, 2019), with a high ratio indicating increased free fatty acid production, an increase in blood lipid levels, and fat accumulation (Xue et al., 2016; Leustean et al., 2018). The data revealed that the F/B ratio was significantly and positively correlated with BW, which was similar to the findings of recent studies with overweight people (Larsen et al., 2010; Schwiertz et al., 2010), though the findings differed from those of other studies (Duncan et al., 2008). Therefore, in this study, a decrease in the F/B ratio may have been responsible for the weight loss of diabetes model rats, indicating a link between diabetes and differences in gut microbial communities. According to our findings, Chao1 diversity decreased with BW, which was most likely due to a positive relationship between BW and the F/B ratio (Supplementary Figure 6). Our findings suggested that the XC + MET combination can influence fat accumulation by regulating the ratio of the dominant flora in the rat intestine.

By comparing bacterial genera, we were able to determine how MET and XC affected gut microbial communities. We discovered that MET and XC could restore the abundance of gut microbial genera in diabetes, specifically the abundance of *Lactobacillus*, *Prevotella*, Bacteroidetes, *Blautia*, and *Ruminococcus*. *Prevotella* and *Bacteroides* were significantly inhibited, but *Ruminococcus* were promoted (Figure 3C). *Blautia* is a key Short Chain Fatty Acid (SCFA)-producing strain that has anti-inflammatory effects (Benítez-Páez et al., 2020), which is beneficial for metabolic diseases (Rodriguez et al., 2020). It is also negatively related to visceral fat levels (Ozato et al., 2019). The abundance of *Blautia* was shown to increase in certain disorders such as non-alcoholic steatohepatitis (Del Chierico et al., 2016) and diabetes (Egshatyan et al., 2016; Wei et al., 2018), which was similar to the results of this study. Additionally, its abundance was lower in the XC + MET-treated rats relative to that in the diabetic rats. Furthermore, the relationship of *Blautia* abundance with certain diabetes-associated metabolic indicators was analyzed, and the results indicated that after XC + MET treatment, the abundance of *Blautia* was significantly positively related to NLRP3, PBG, and liver weight/BW and significantly negatively related to FINS and diabetes-related lipid metabolism such as fatty acid biosynthesis. This might be because XC + MET promoted the activation of the anti-inflammatory pathway and regulated metabolic homeostasis. This correlation data suggests that *Blautia* promotes metabolic disorders. Therefore, the decrease in *Blautia* abundance observed in XC + MET-treated rats (Figures 2E, 3C,D) would be expected to have a positive effect on diabetic rats. However, more studies should be conducted to confirm the function of *Blautia*.

In this study, we also observed *Bacteroidetes* and *Prevotella* enrichment in diabetic rats, which decreased when XC + MET was administered. The abundance of *Bacteroidetes* and *Prevotella* were significantly negatively associated with the metabolism of carbohydrates, such as pyruvate, propionate, and butanoate metabolism. Pyruvate mainly comes from carbohydrates and is the end product of the glycolysis pathway, which has a key effect on the metabolic relationship of sugar and amino acids with fats and can further be metabolized to SCFAs (Oliphant and Allen-Vercoe, 2019). The elevated synthesis of SCFA can exert anti-obesity and anti-diabetic effects (Pedersen et al., 2016; Pingitore et al., 2017). *Prevotella* sp. in the gut may positively or negatively affect human health. Some researchers found that *Prevotella* had a beneficial effect, and high *Prevotella* abundance could promote glycogen storage and induce glucose intolerance (Kovatcheva-Datchary et al., 2015). However, other researchers found that *Prevotella* strains might increase the occurrence of diabetes by promoting chronic inflammation (Larsen, 2017; De Filippis et al., 2019), as found in this study. We found that the intervention of XC + MET could affect the high abundance of *Prevotella* in diabetic rats.

Dysfunctional intestinal microflora can stimulate intestinal wall cells to secrete 5-serotonin by secreting secondary bile acids, thereby increasing blood glucose levels, which maintains a continuous hyperglycemic state in the patients, leading to the development and progression of diabetes (Martin et al., 2019). *Lactobacillus* can regulate the glucose-sensing machinery associated with additional pathways (Yun et al., 2009; Lin et al., 2014). The secondary bile acids can act as regulators for the Triglyceride (TG) and glucose homeostasis in the host, which may be the therapeutic targets of the anti-metabolic disorder (Kuno et al., 2018). In our study, *Lactobacillus* abundance, which was related negatively to secondary bile acid biosynthesis and the insulin signaling pathway, exhibited significant recovery from dysbiosis after treatment with the XC + MET combination, thus suggesting that the XC + MET interventional biomarker had certain effects. Additionally, the abundance of *Lactobacillus* showed a positive correlation with the metabolism of amino acids, such as arginine/proline metabolism, glycine/threonine/serine metabolism, and valine/leucine/isoleucine biosynthesis. The glycine level was the most significantly related to the enhanced insulin sensitivity, as reported in previous studies (Gall et al., 2010; Sekhar et al., 2011).

Several studies have shown that the gut microbial community has metabolic activities and functions. Moreover, their alterations can affect host metabolism (Tanca et al., 2017). Therefore, this study analyzed the relationship of the metabolic activity (predicted by the 16S rDNA sequence) with host metabolites (determined by metabolomics) based on the KEGG database from fecal samples. The results showed that the gut microbial function and composition substantially overlapped and differed in the diabetic rats compared to those in the healthy controls. We identified the genus *Lactobacillus* and carbohydrate metabolism, as well as the insulin signaling pathway, as biomarkers and candidate therapeutic targets for diabetes. A high level of carbohydrate consumption might be related to the diabetes pathogenic mechanism. Excess carbohydrate consumption can induce fat deposition in the body, elevate blood glucose levels, and cause a greater burden on islet cells. Additionally, long-term accumulation can substantially increase diabetes risk. Based on our results, XC + MET treatment affected various biological activities, such as the metabolism of carbohydrates, amino acids, and lipids and the insulin signaling pathway.

Finally, our findings suggested that XC could assist MET by improving postprandial hyperglycemia, shaping the microbiome, and regulating carbohydrate, amino acid, and lipid metabolism. In particular, the insulin signaling pathway has implications for the pathogenesis of diabetes. Therefore, we hypothesized that the effects of XC on diabetic rats were closely related to SCFA-producing and anti-inflammatory bacteria. The metabolites of which could improve intestinal barrier function and gut permeability, inhibit inflammation, and thus ameliorate IR and attenuate diabetes. Based on the previous research, it was proposed that modifying gut microbiota could be one of the proposed mechanisms that XC can auxiliary MET to treat T2DM. Our findings shed light on the roles that XC played in adjunct hypoglycemic activities from the perspective of the gut microbiota, which could help further clarify its anti-diabetic mechanism *in vivo* and effectively apply to clinical practice in treating diabetes.

There are several limitations to our study. At first, this study lacks relevant pathological research on pancreatic tissue and cannot fully comprehend the pathological changes of pancreatic islets during the diabetes process. The OGTT is the gold standard for diagnosing diabetes. Due to the complexities of requiring multiple blood samples, only FPG and 2 h-PG levels were measured, and the area under the insulin curve during glucose tolerance was not monitored. To better understand the synergistic effect of XC, it is necessary to track the dynamic changes in islets as diabetes progresses.

## Data availability statement

The data presented in this study are deposited in the NCBI repository, accession number: PRJNA818640.

## Ethics statement

This animal study was reviewed and approved by the Care and Use of Laboratory Animals (No. 2018-06013).

## Author contributions

XG, YN, and LL were responsible for conceptualizing the study, designing the experiments, and interpreting the results. YQ, and MS were in charge of conducting the experiments and analyzing the data. XF and WZ were responsible for generating graphs. YJ, WS, and QD were in charge of conceptualizing the study and interpreting the results. XG, CW, and RZ contributed to the manuscript writing. XH analyzed the data, edited the manuscript, and made outstanding contributions in the process of revising the manuscript. All authors approved the final version of the manuscript.

## Footnotes

1
http://huttenhower.sph.harvard.edu/galaxy
