# Supplementary material for: Scrophulariae Radix-Atractylodes sinensis pair and metformin inhibit inflammation by modulating gut microbiota of high-fat diet/streptozotocin-induced diabetes in rats
Source: Front Microbiol. 2022 Nov 30;13:900021. doi: 10.3389/fmicb.2022.900021 (PMC9748418; doi:10.3389/fmicb.2022.900021)
Supplement: Supplementary file 1 [file Data_Sheet_1.docx]

Supplementary Material


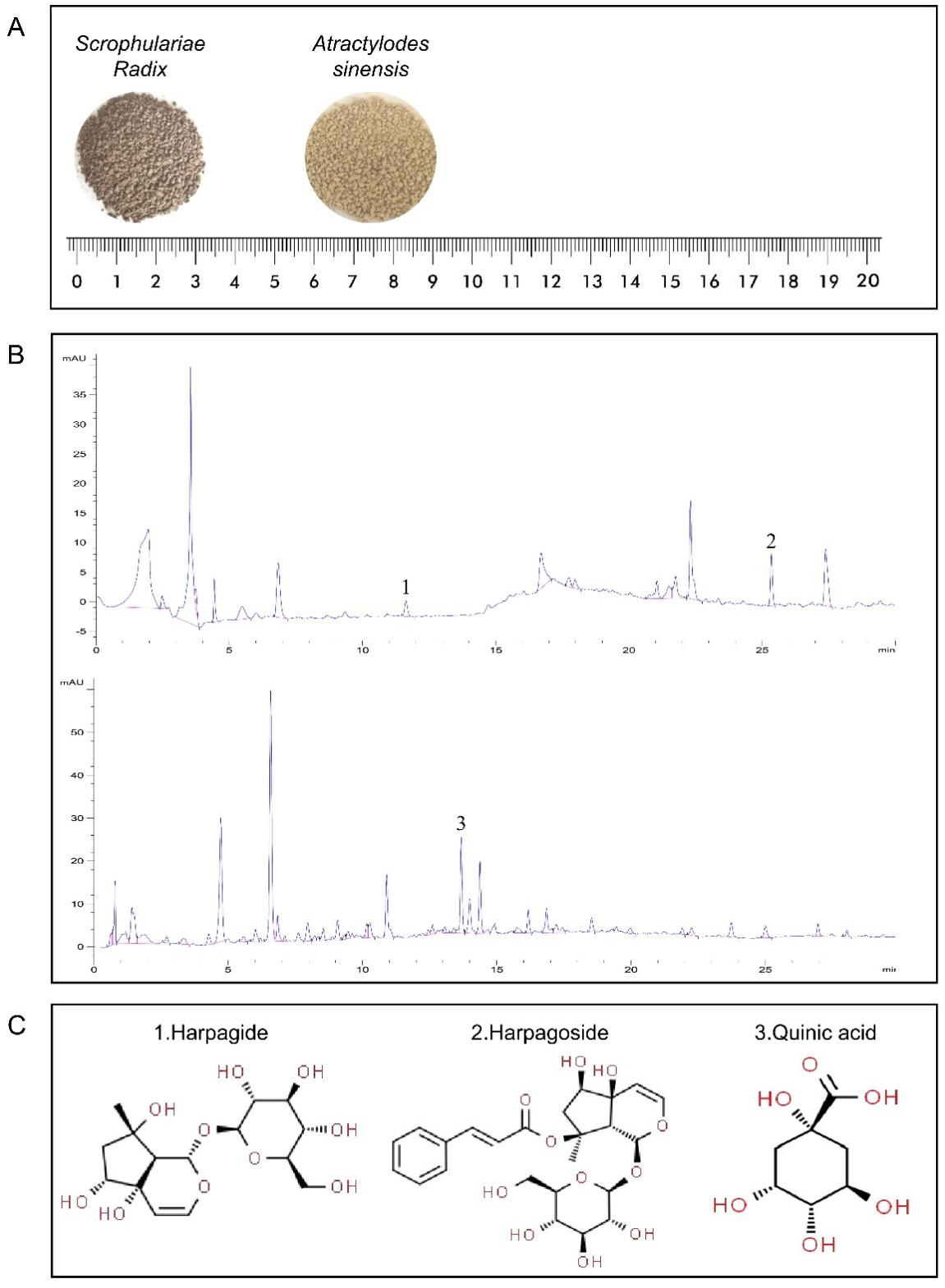


Supplementary Figure 1. The XC pair herbal formula. (a) The images show unboiled granules from two herbal medicines contained in the XC herbal formula. (b) HPLC analyses of Scrophulariae Radix and Atractylodes sinensis. (c) The three main chemical compounds of the XC pair (the structural formula of each compound was cited from <http://www.>chemspider. com/)


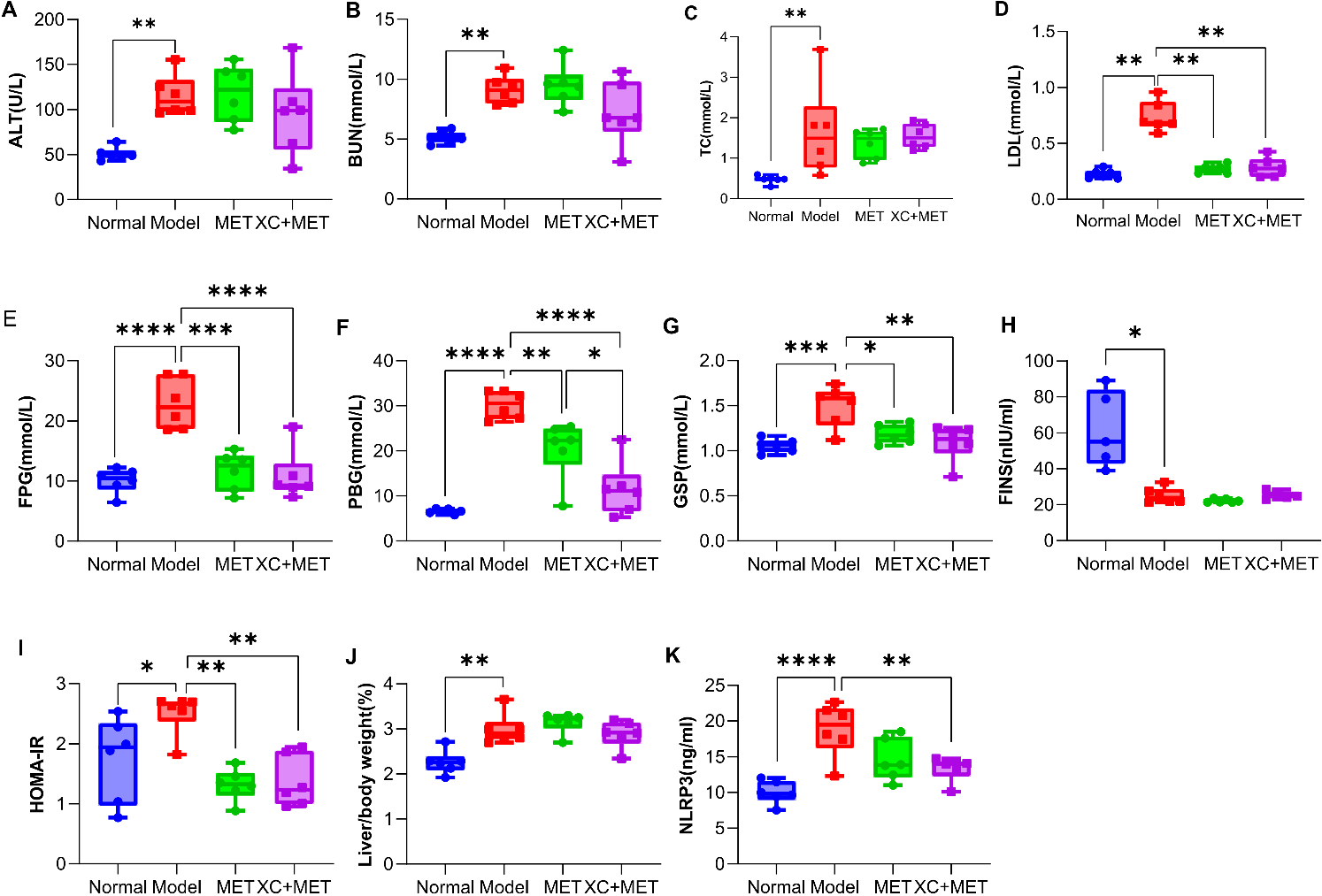


**Supplementary Figure 2.** The effects of the XC pair and MET on liver and kidney function, and glucose and lipid homeostasis in the T2DM rats; (A) ALT, (B) BUN, (C) TC, (D) LDL, (E) FPG, (F) PBG, (G) GSP, (H) FINS, (I) HOMA-IR, (J) liver weight/body weight, and (K) NLRP3. MET: Metformin, XC+MET: Combination of the Scrophulariae Radix and Atractylodes sinensis pair. Data are displayed as the mean ±SD, n = 6; *P < 0.05, **P < 0.01, ***P < 0.001, and ****P < 0.0001.


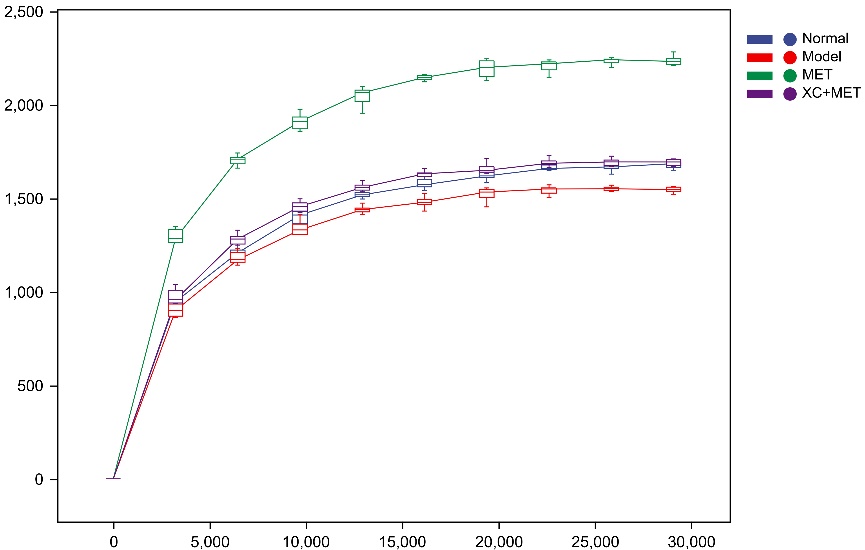


**Supplementary Figure 3.** The rarefaction curves obtained by rarefaction analyses of the 16S rRNA V3-V4 pyrosequencing tags for the gut microbial communities; n = 6.


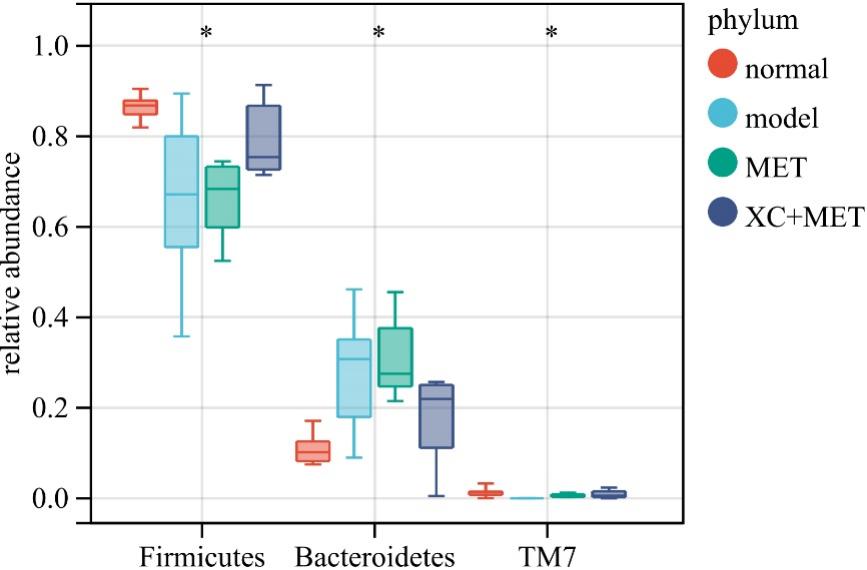


**Supplementary Figure 4.** The abundance of various phyla across the four groups. The level of significance was determined by performing Wilcoxon’s signed-rank test; *P < 0.05, **P < 0.01, and ***P < 0.001.


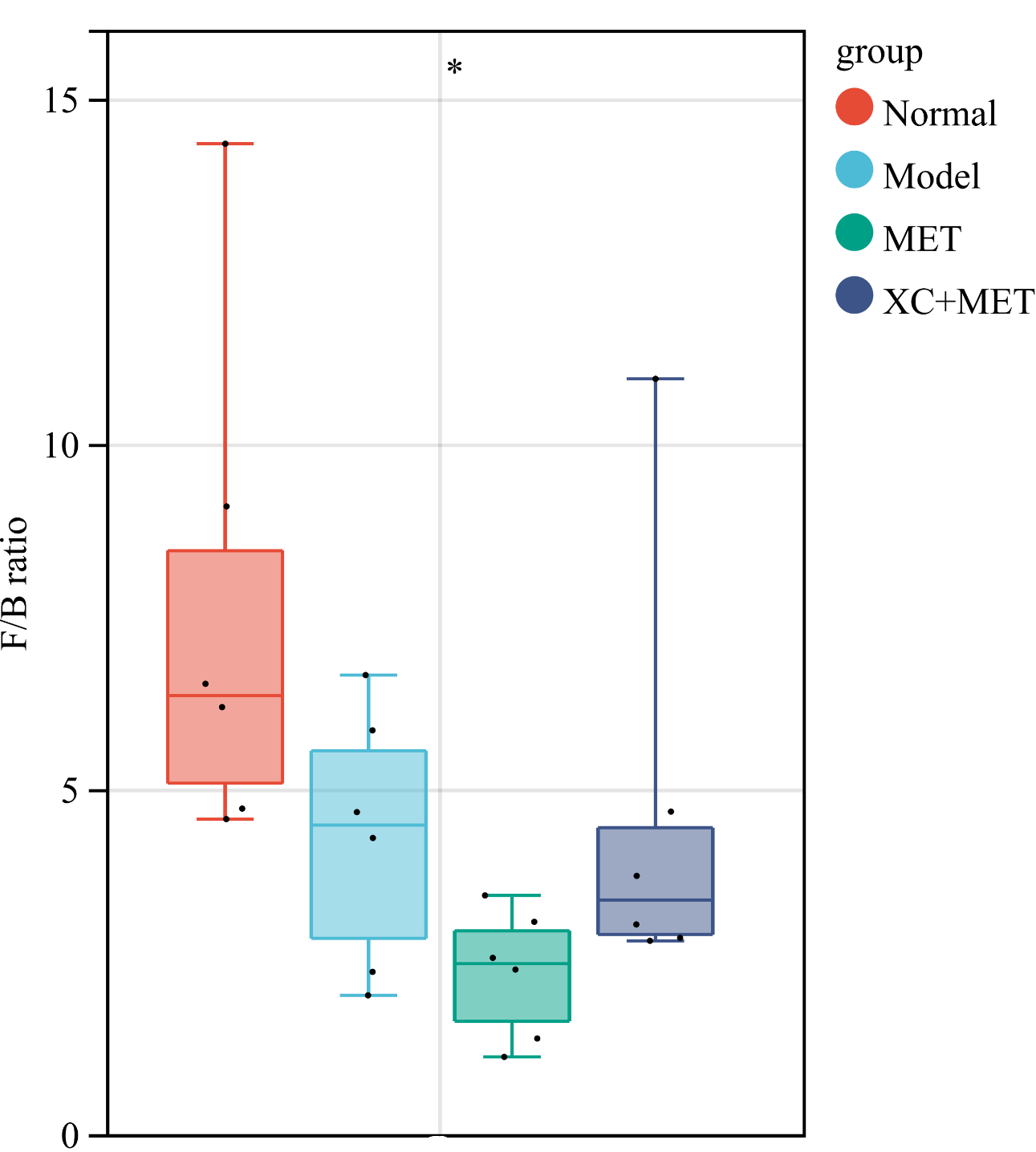


**Supplementary Figure 5.** The Firmicutes-to-Bacteroidetes (F/B) ratios among the four groups. The level of significance among the groups was determined by performing Wilcoxon’s signed-rank test; *P < 0.05, **P < 0.01, and ***P < 0.001.


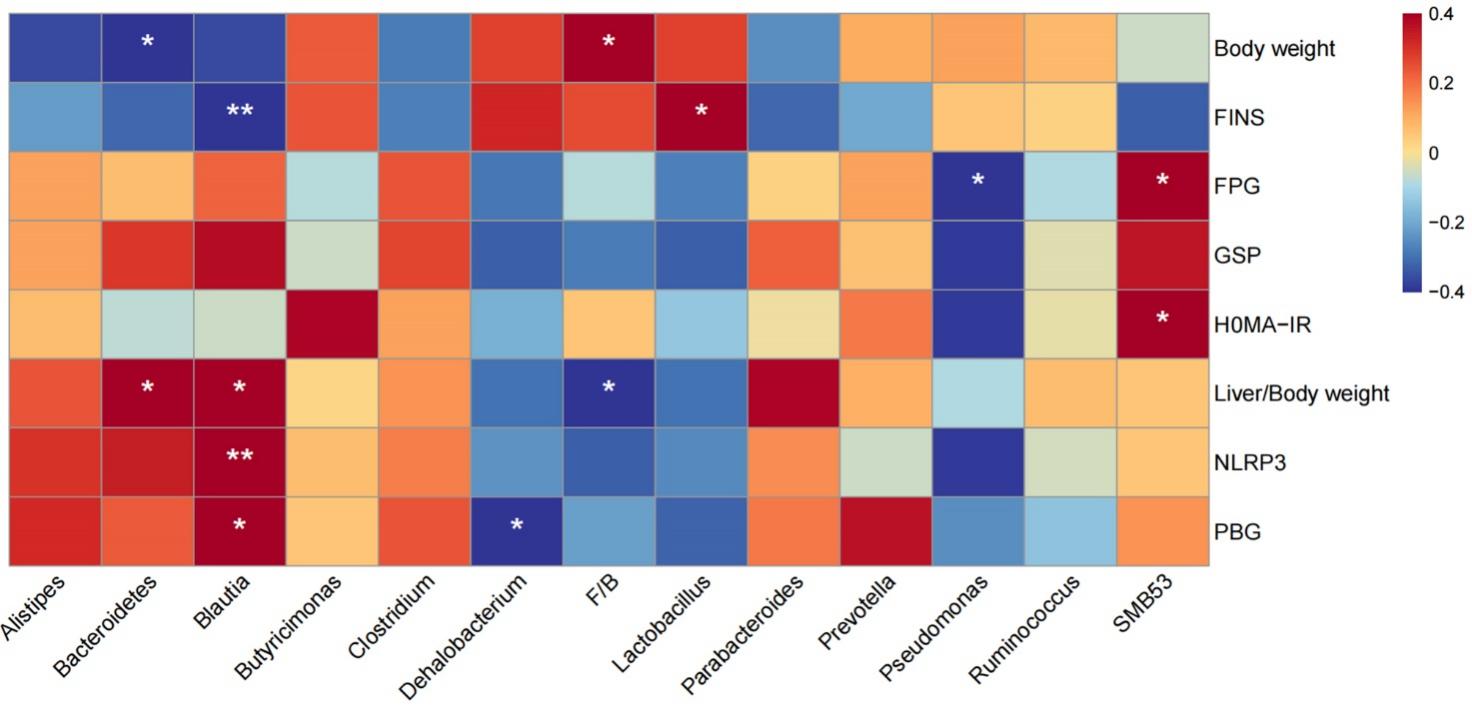


**Supplementary Figure 6.** The correlation between microbial biomarkers and biochemical indicators. The panel shows the relationship of the F/B ratio with 12 different genera with body-weight, FINS, FPG, PBG, GSP, HOMA-IR, liver weight/body weight, and NLRP3. The color intensity indicates the correlation strength (red and blue indicate positive and negative correlation, respectively); *P < 0.05, **P < 0.01, ***P < 0.001, and ****P < 0.0001; n = 6.

Supplementary Table 1. The components of XC herbal formula

| Herbal name | Botanical Latin name | Place of origin | Boil-free granules weight (g/day) *^a^* |
| --- | --- | --- | --- |
| Xuan-Shen | Scrophulariae Radix | Jiangyin | 6 |
| Cang-Zhu | Atractylodes sinensis | Jiangyin | 3 |

*^a^* Each herb was processed into boil-free granules and packed into individual bags.

Supplementary Table 2. The chemical composition detected of Scrophulariae Radix sample by HPLC.

| Serial  number | Retention time  (min) | Mass spectrum information | | | | |
| --- | --- | --- | --- | --- | --- | --- |
|  |  | Type | Peak width  (min) | Peak area  (mAu*s) | Peak height  (mAu) | Peak area  (%) |
| 1 | 0.632 | BV | 0.0589 | 10.16127 | 2.63907 | 0.5146 |
| 2 | 0.691 | VV | 0.042 | 9.11750 | 3.12619 | 0.4617 |
| 3 | 0.789 | VB | 0.0526 | 51.50970 | 14.80129 | 2.6086 |
| 4 | 1.187 | BV | 0.1814 | 40.43095 | 2.81696 | 2.0476 |
| 5 | 1.421 | VV | 0.1393 | 87.38710 | 8.40571 | 4.4256 |
| 6 | 1.881 | VB | 0.2903 | 48.98792 | 2.09916 | 2.4809 |
| 7 | 2.709 | BB | 0.0989 | 9.84601 | 1.53402 | 0.4986 |
| 8 | 3.374 | BB | 0.1578 | 16.37244 | 1.44922 | 0.8292 |
| 9 | 4.282 | BB | 0.1051 | 16.76825 | 2.47296 | 0.8492 |
| 10 | 4.728 | BB | 0.1058 | 201.80013 | 28.79716 | 10.2198 |
| 11 | 5.576 | BB | 0.1062 | 8.47495 | 1.23357 | 0.4292 |
| 12 | 6.014 | BV | 0.1029 | 21.12216 | 2.90390 | 1.0697 |
| 13 | 6.589 | BV R | 0.0872 | 337.78854 | 58.63319 | 17.1067 |
| 14 | 6.844 | VV E | 0.0797 | 33.08967 | 6.05616 | 1.6758 |
| 15 | 7.108 | VB | 0.0634 | 5.16707 | 1.27135 | 0.2617 |
| 16 | 7.613 | BB | 0.113 | 15.20269 | 2.03871 | 0.7699 |
| 17 | 7.962 | BB | 0.106 | 30.00578 | 4.37693 | 1.5196 |
| 18 | 8.226 | BV | 0.1113 | 7.34643 | 1.08138 | 0.3720 |
| 19 | 8.541 | VB | 0.0918 | 17.26867 | 2.80769 | 0.8745 |
| 20 | 9.079 | VB R | 0.1032 | 32.00457 | 4.59805 | 1.6208 |
| 21 | 9.328 | BV | 0.0735 | 5.42869 | 1.14040 | 0.2749 |
| 22 | 9.461 | VV | 0.0902 | 10.22087 | 1.69973 | 0.5176 |
| 23 | 10.162 | BV | 0.0843 | 18.81535 | 3.31206 | 0.9529 |
| 24 | 10.277 | VB | 0.1063 | 23.75001 | 3.36682 | 1.2028 |
| 25 | 10.900 | BB | 0.0871 | 87.74991 | 14.82517 | 4.4439 |
| 26 | 12.449 | BV | 0.0785 | 6.46998 | 1.24841 | 0.3277 |
| 27 | 12.611 | VB | 0.0785 | 11.09650 | 2.13978 | 0.5620 |
| 28 | 13.072 | VB | 0.0899 | 7.37007 | 1.34606 | 0.3732 |
| 29 | 13.407 | BV E | 0.0877 | 7.18446 | 1.20217 | 0.3638 |
| 30 | 13.678 | VB R | 0.0762 | 111.34569 | 22.32670 | 5.6389 |
| 31 | 13.993 | BV R | 0.1005 | 53.80988 | 8.00144 | 2.7251 |
| 32 | 14.377 | BV R | 0.0752 | 83.71243 | 17.08031 | 4.2395 |
| 33 | 14.665 | VV E | 0.1086 | 7.97799 | 1.02699 | 0.404 |
| 34 | 14.929 | VB | 0.103 | 16.96913 | 2.22428 | 0.8594 |
| 35 | 15.753 | BB | 0.143 | 13.32737 | 1.30700 | 0.6749 |
| 36 | 16.172 | BB | 0.0766 | 27.12654 | 5.40077 | 1.3738 |
| 37 | 16.853 | BV R | 0.1013 | 40.16555 | 5.62791 | 2.0341 |
| 38 | 17.219 | VV | 0.1188 | 14.47395 | 1.86051 | 0.7330 |
| 39 | 17.444 | VV | 0.1044 | 9.28079 | 1.25288 | 0.4700 |
| 40 | 18.531 | BB | 0.0957 | 19.72203 | 3.20980 | 0.9988 |
| 41 | 19.454 | VB | 0.0879 | 6.37538 | 1.12787 | 0.3229 |
| 42 | 19.984 | BB | 0.1211 | 11.30077 | 1.25436 | 0.5723 |
| 43 | 21.904 | BB | 0.082 | 7.77513 | 1.51242 | 0.3938 |
| 44 | 22.253 | BB | 0.1126 | 13.19997 | 1.73894 | 0.6685 |
| 45 | 23.734 | BB | 0.1092 | 24.62436 | 3.37349 | 1.2471 |
| 46 | 25.000 | BB | 0.1127 | 21.40328 | 2.88038 | 1.0839 |
| 47 | 26.962 | BB | 0.0854 | 15.83333 | 2.82349 | 0.8019 |
| 48 | 28.038 | BB | 0.0734 | 6.97224 | 1.46816 | 0.3531 |
| 49 | 30.113 | BB | 0.0702 | 12.12289 | 2.70770 | 0.6139 |
| 50 | 31.760 | BB | 0.1082 | 17.10083 | 2.21265 | 0.8660 |
| 51 | 32.574 | BV | 0.0904 | 14.83574 | 2.32523 | 0.7513 |
| 52 | 33.262 | BB | 0.14 | 29.47661 | 3.01756 | 1.4928 |
| 53 | 38.957 | BB | 0.0929 | 8.54234 | 1.44670 | 0.4326 |
| 54 | 39.379 | BB | 0.0997 | 8.38898 | 1.29287 | 0.4248 |
| 55 | 40.576 | BB | 0.1003 | 65.71316 | 9.54786 | 3.3279 |
| 56 | 41.483 | BB | 0.105 | 135.08214 | 19.44481 | 6.8410 |

Supplementary Table 3. The chemical composition detected of Atractylodes sinensis sample by HPLC.

| Serial  number | Retention time  (min) | Mass spectrum information | | | | |
| --- | --- | --- | --- | --- | --- | --- |
|  |  | Type | Peak width  (min) | Peak area  (mAu*s) | Peak height  (mAu) | Peak area  (%) |
| 1 | 1.957 | BV | 0.4031 | 433.70404 | 13.34884 | 25.6667 |
| 2 | 2.487 | VB | 0.1049 | 14.23818 | 2.16076 | 0.8426 |
| 3 | 3.550 | BV R | 0.1045 | 345.30414 | 43.55754 | 20.4352 |
| 4 | 3.743 | VB E | 0.0877 | 16.49389 | 2.92961 | 0.9761 |
| 5 | 4.438 | BB | 0.0620 | 30.49002 | 7.41072 | 1.8044 |
| 6 | 5.485 | BB | 0.2190 | 34.36991 | 2.24042 | 2.0340 |
| 7 | 6.835 | BB | 0.1450 | 86.93372 | 9.30175 | 5.1448 |
| 8 | 11.633 | BB | 0.1197 | 21.15721 | 2.69274 | 1.2521 |
| 9 | 16.711 | BB | 0.1877 | 77.95541 | 5.91890 | 4.6134 |
| 10 | 17.761 | BV | 0.1372 | 12.95727 | 1.52406 | 0.7668 |
| 11 | 17.985 | VB | 0.1147 | 11.33980 | 1.45900 | 0.6711 |
| 12 | 21.050 | BB | 0.1354 | 29.37225 | 2.96829 | 1.7383 |
| 13 | 21.498 | BV | 0.1854 | 25.85861 | 2.01957 | 1.5303 |
| 14 | 21.754 | VB | 0.1395 | 36.64279 | 3.70040 | 2.1685 |
| 15 | 22.318 | BB | 0.0997 | 113.02028 | 16.54997 | 6.6886 |
| 16 | 25.350 | BB | 0.0826 | 47.32744 | 8.81501 | 2.8008 |
| 17 | 27.389 | BB | 0.1262 | 83.86355 | 9.57306 | 4.9631 |
| 18 | 32.125 | BB | 0.1735 | 36.84220 | 3.12653 | 2.1803 |
| 19 | 33.212 | BB | 0.1821 | 41.36425 | 3.34815 | 2.4479 |
| 20 | 35.238 | BB | 0.1460 | 130.92235 | 13.40342 | 7.7480 |
| 21 | 36.656 | BBA | 0.1829 | 59.59805 | 4.73318 | 3.5270 |

Supplementary Table 4. The LDA score and P-value of genus that changed significantly during the development of T2DM and formed a significant difference from Normal group.

|  | Phylum | Genus | LDA_score | Pvalue |
| --- | --- | --- | --- | --- |
| Gut microbiota increased gradually with the  development of T2DM | Actinobacteria | Bifidobacterium | 4.448 | 0.010 |
|  | Bacteroidetes | Paraprevotella | 3.025 | 0.021 |
|  | Bacteroidetes | Bacteroides | 4.111 | 0.010 |
|  | Bacteroidetes | Prevotella | 4.701 | 0.004 |
|  | Firmicutes | Phascolarctobacterium | 3.441 | 0.010 |
| Gut microbiota reduced  gradually with the  development of T2DM | Actinobacteria | Adlercreutzia | 3.187 | 0.016 |
|  | Firmicutes | Pediococcus | 3.759 | 0.016 |
|  | Firmicutes | Clostridium | 3.432 | 0.037 |
|  | Firmicutes | Dehalobacterium | 3.334 | 0.020 |
|  | Firmicutes | Anaerostipes | 3.673 | 0.002 |
|  | Firmicutes | Ruminococcus | 3.634 | 0.016 |

Supplementary Table 5. The LDA score and P-value of genus that changed significantly during the development of T2DM and formed a significant difference from MET-treated group.

|  | Phylum | Genus | LDA_score | Pvalue |
| --- | --- | --- | --- | --- |
| Gut microbiota increased gradually with the development of MET | Proteobacteria | Pseudomonas | 3.490 | 0.003 |
|  | Firmicutes | Allobaculum | 3.287 | 0.016 |
|  | Firmicutes | Ruminococcus | 4.312 | 0.004 |
|  | Firmicutes | Butyricicoccus | 3.824 | 0.013 |
|  | Firmicutes | Clostridium | 3.544 | 0.008 |
|  | Firmicutes | Dehalobacterium | 3.648 | 0.014 |
|  | Firmicutes | SMB53 | 3.258 | 0.010 |
|  | Firmicutes | Clostridium | 4.268 | 0.004 |
|  | Firmicutes | ~~02d06~~ | 3.845 | 0.007 |
|  | Firmicutes | Turicibacter | 3.839 | 0.004 |
|  | Firmicutes | Streptococcus | 3.877 | 0.010 |
|  | Actinobacteria | Adlercreutzia | 3.333 | 0.016 |
| Gut microbiota reduced gradually with the development of MET | Firmicutes | Coprobacillus | 4.065 | 0.022 |
|  | Firmicutes | Lactobacillus | 5.257 | 0.010 |
|  | Bacteroidetes | Prevotella | 4.669 | 0.004 |
|  | Bacteroidetes | Bacteroides | 4.058 | 0.037 |

Supplementary Table 6. The LDA score and P-value of genus that changed significantly during the development of T2DM and formed a significant difference from XC+MET-treated group.

|  | Phylum | Genus | LDA_score | Pvalue |
| --- | --- | --- | --- | --- |
| Gut microbiota increased  gradually with the  development of XC+MET | Firmicutes | Streptococcus | 3.679 | 0.016 |
|  | Firmicutes | Clostridium | 3.852 | 0.010 |
|  | Firmicutes | Dehalobacterium | 3.080 | 0.031 |
|  | Firmicutes | Coprococcus | 3.251 | 0.037 |
|  | Firmicutes | Ruminococcus | 4.308 | 0.016 |
|  | Firmicutes | Allobaculum | 3.172 | 0.010 |
|  | Proteobacteria | Pseudomonas | 3.731 | 0.031 |
| Gut microbiota reduced  gradually with the  development of XC+MET | Actinobacteria | Bifidobacterium | 4.474 | 0.010 |
|  | Bacteroidetes | Butyricimonas | 3.586 | 0.031 |
|  | Bacteroidetes | Paraprevotella | 3.033 | 0.020 |
|  | Bacteroidetes | Bacteroides | 4.108 | 0.010 |
|  | Bacteroidetes | Prevotella | 4.720 | 0.004 |
|  | Firmicutes | Phascolarctobacterium | 3.417 | 0.025 |
|  | Firmicutes | Coprobacillus | 3.602 | 0.022 |

Supplementary Table 7. The LDA score and P-value of genus with significant difference between XC+MET and MET groups.

|  | Phylum | Genus | LDA_score | Pvalue |
| --- | --- | --- | --- | --- |
| Gut microbiota increased gradually with the  development of XC+MET | Firmicutes | Pediococcus | 4.570 | 0.025 |
| Gut microbiota reduced gradually with the  development of XC+MET | Firmicutes | Butyricicoccus | 4.554 | 0.033 |
|  | Firmicutes | SMB53 | 4.119 | 0.037 |
